# Clustered Coding Variants in the Glutamate Receptor Complexes of Individuals with Schizophrenia and Bipolar Disorder

**DOI:** 10.1371/journal.pone.0019011

**Published:** 2011-04-29

**Authors:** René A. W. Frank, Allan F. McRae, Andrew J. Pocklington, Louie N. van de Lagemaat, Pau Navarro, Mike D. R. Croning, Noboru H. Komiyama, Sophie J. Bradley, R. A. John Challiss, J. Douglas Armstrong, Robert D. Finn, Mary P. Malloy, Alan W. MacLean, Sarah E. Harris, John M. Starr, Sanjeev S. Bhaskar, Eleanor K. Howard, Sarah E. Hunt, Alison J. Coffey, Venkatesh Ranganath, Panos Deloukas, Jane Rogers, Walter J. Muir, Ian J. Deary, Douglas H. Blackwood, Peter M. Visscher, Seth G. N. Grant

**Affiliations:** 1 Wellcome Trust Sanger Institute, Genome Campus, Hinxton, Cambridgeshire, United Kingdom; 2 Queensland Institute of Medical Research, Royal Brisbane Hospital, Brisbane, Australia; 3 School of Informatics, Edinburgh University, Edinburgh, United Kingdom; 4 MRC Human Genetics, Institute of Genetics and Molecular Medicine, Western General Hospital, Edinburgh, United Kingdom; 5 Division of Psychiatry, University of Edinburgh, Royal Edinburgh Hospital, Edinburgh, United Kingdom; 6 Department of Psychology, Centre for Cognitive Ageing and Cognitive Epidemiology, University of Edinburgh, Edinburgh, United Kingdom; 7 Department of Cell Physiology and Pharmacology, University of Leicester, Leicester, United Kingdom; University of Queensland, Australia

## Abstract

Current models of schizophrenia and bipolar disorder implicate multiple genes, however their biological relationships remain elusive. To test the genetic role of glutamate receptors and their interacting scaffold proteins, the exons of ten glutamatergic ‘hub’ genes in 1304 individuals were re-sequenced in case and control samples. No significant difference in the overall number of non-synonymous single nucleotide polymorphisms (nsSNPs) was observed between cases and controls. However, cluster analysis of nsSNPs identified two exons encoding the cysteine-rich domain and first transmembrane helix of GRM1 as a risk locus with five mutations highly enriched within these domains. A new splice variant lacking the transmembrane GPCR domain of GRM1 was discovered in the human brain and the GRM1 mutation cluster could perturb the regulation of this variant. The predicted effect on individuals harbouring multiple mutations distributed in their ten hub genes was also examined. Diseased individuals possessed an increased load of deleteriousness from multiple concurrent rare and common coding variants. Together, these data suggest a disease model in which the interplay of compound genetic coding variants, distributed among glutamate receptors and their interacting proteins, contribute to the pathogenesis of schizophrenia and bipolar disorders.

## Introduction

Schizophrenia and bipolar disorder are common heritable disorders showing considerable clinical and genetic overlap [1] for which a neurobiological explanation remains wanting. Pharmacological studies suggest at least two molecular models: the ‘glutamate hypothesis’, arising from observations that the NMDA (*N*-methyl-D-aspartate) receptor antagonists produce schizophrenia-like psychotic symptoms [2], [3] and the ‘dopamine hypothesis’, based on the discovery that dopaminergic antagonists are anti-psychotics [4]. However, it is becoming apparent that glutamate and dopamine receptors are functionally associated within complexes, particularly via scaffold proteins [5], [6], connecting these hypotheses and highlighting the potential importance of post-synaptic signalling proteins in these disorders.

Recent genome-wide association studies (GWAS) of schizophrenia identified rare *de novo* copy number variation (CNV) [7] and an increased load of micro-deletions and micro-duplications around coding regions [8], [9]. Although the causative variants are not yet known, an hypothesis is emerging from these studies that it is unlikely the disease is represented by a limited number of common variants. Instead, the loci identified contain rare variants predicted to affect a disparate selection of genes, and suggesting multiple ‘routes’ for the aetiology of the disease [10]. Thus far, genome-wide screens have not addressed the question of whether rare coding single nucleotide polymorphisms (nsSNPs) might contribute to psychiatric diseases, since they are undetectable by CNV scans and are not represented on SNP arrays [11].

The function of both ion-channel forming (ionotropic) [12], [13] and G-protein coupled (metabotropic) [14], [15] glutamate receptors is in large part dependent on their physical interactions with intracellular scaffold proteins, including the membrane-associated guanylate kinase (MAGUK) family. Proteomic studies show that these receptors and scaffold proteins assemble into complexes with a diverse range of enzymes, cytoskeletal and other proteins [16], [17]. The MAGUK-associated signalling complexes (MASCs) comprise 100–200 different proteins that in addition to binding glutamate receptors interact with a much larger sub-cellular architecture, the post-synaptic density (PSD) [17], [18], [19]. Central in this complex are 10 hub genes that are defined on the basis of predicted protein-protein interaction data and appear to coordinate the complex mechanisms of these receptors (Supporting Information S1) [20]. These 10 genes are subunits of the ionotropic NMDA receptor (*GRIN1*, *GRIN2A*, *GRIN2B*), AMPA (α-amino-3-hydroxy-5-methyl-4-isoxazolepropionic acid) receptor (*GRIA1*, *GRIA2*), metabotropic (*GRM1*) glutamate receptor, and their MAGUK primary binding partners *DLG1*, *DLG2*, *DLG3*, and *DLG4*, which encode SAP97, PSD93, SAP102 and PSD95, respectively.

**Figure 1:**
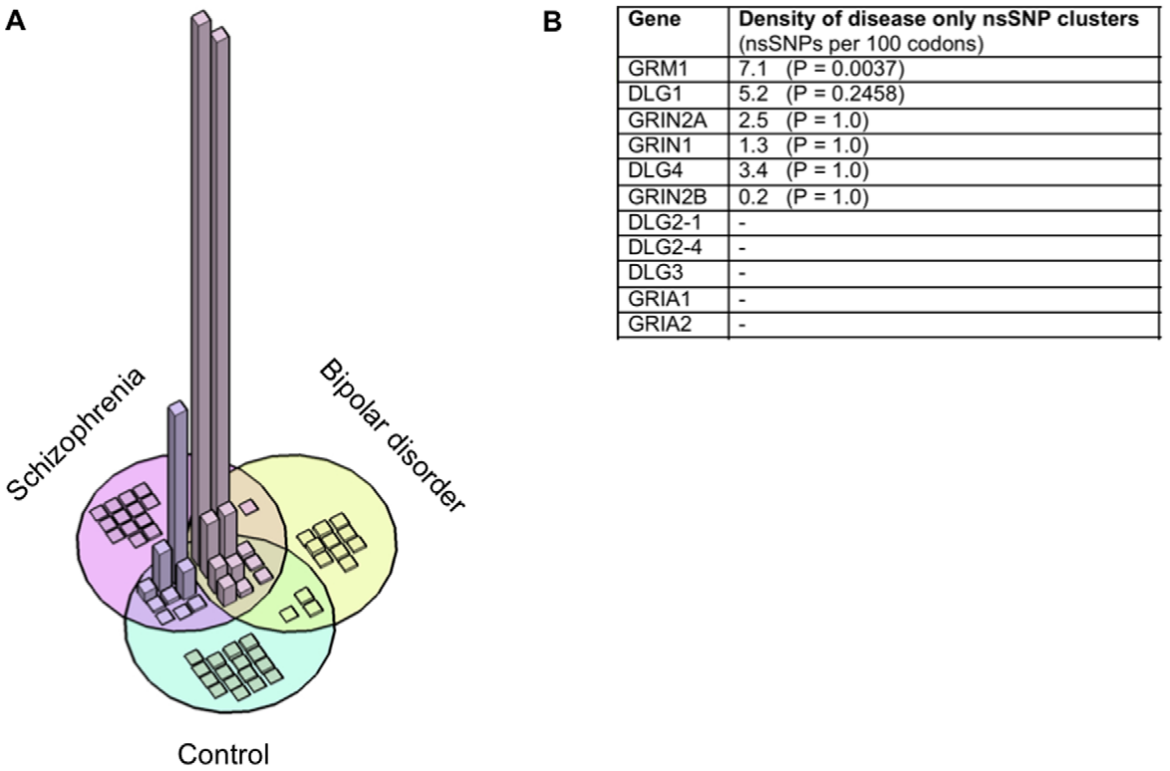
The frequency and clustering of nsSNPs. **A)** 3-dimensional Venn diagram showing the relative frequency (height) of 62 different non-synonymous single nucleotide polymorphisms (nsSNPs; represented as blocks) from exon re-sequencing of schizophrenic, bipolar and controls. All data points are in view and range from less than 0.1% to 45% minor allele frequency. NsSNPs found only in one of the cohorts exhibit very low frequency compared to the common variants found in all (see supplementary datasheet S2, columns I, K and M). **B)** Table showing the exon re-sequenced genes ranked by the significance of nsSNP density of their disease only nsSNP clusters. Disease only nsSNPs are variants that are found at least once in one of the disease cohorts and were excluded from the control cohort. *DLG2* is represented by two splice variants, in which nsSNPs were found. nsSNP density is defined as the number of nsSNP per 100 codons (or residues). nsSNP clusters were identified computationally and P-values were calculated by randomization (see Supporting Information S1).

To address the possibility that glutamate receptors together with their associated signalling proteins, acting alone or in combination, contribute to genetic susceptibility in schizophrenia and bipolar disorder, a study of rare and common coding polymorphisms was performed. Variants that alter the amino acid sequence, the protein structure and may be functionally important were identified by deep sequencing exons in the 10 hub genes of 1344 individuals (Figure 1B). These individuals were divided into those with schizophrenia, bipolar disorder and healthy controls.

In a parallel study, we genotyped tag-SNPs within a wider set of 169 MASC genes and 96 other candidate genes. Consistent with other genome wide association studies of schizophrenia and bipolar disorder [10], [21], this latter approach was unable to find any significant common variants with effect on either schizophrenia or bipolar disorder. However, these data provided evidence that the ten genes selected for sequencing showed significantly more association with disease than would be expected by chance (rank sum P = 0.0034, see Supporting Information S1). Overall, this tag-SNP data revealed only marginal association and was unable to identify directly the specific mutations that contribute to disease.

## Results

DNA sequencing of the 10 hub genes from 503 schizophrenic, 263 bipolar and 538 ancestrally matched controls identified 62 different coding variants (non-synonymous single nucleotide polymorphisms; nsSNPs). Of these 62 mutations, 24 were found solely in either the schizophrenia or bipolar disorder cohort and 16 were found solely in the control group (Fisher's exact test, P = 0.39). Since the frequency of these disease only nsSNPs or any single coding polymorphism did not predict disease risk (Figure 1B and Supporting Information S1), we asked whether protein features encoded by these nsSNPs might reveal deficits associated with the disease.

**Figure 2:**
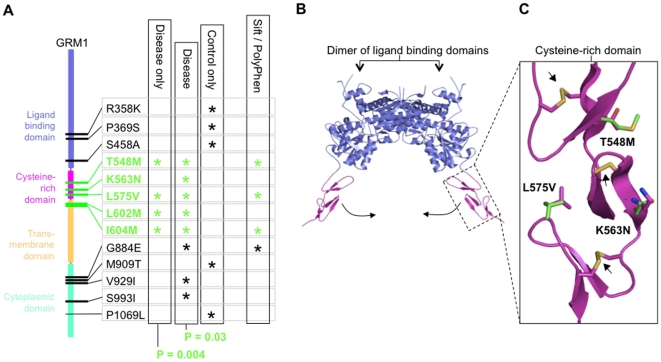
Mapping the *GRM1* nsSNP cluster onto a model of the protein structure. **A)** nsSNP density plot for GRM1 illustrating the distribution along the length of the protein. Each nsSNP is marked by an asterisk if it is found only in a disease cohort (left column), found at least once in the disease and control cohorts (second from left column), found only in the control cohort (third from left column), predicted deleterious by sequence conservation analysis (right column; see Supporting Information S1 and datasheet S3). P-values correspond to the significance of the cluster's (nsSNPs indicated in green) density. **B)** Protein structural model of GRM1 ligand-binding domain (LBD; blue) and cysteine-rich domains (CRD; magenta). The model was generated by Fugue and SCWRL3 using a crystal structure of GRM3 (PDB: 2e4u) as a template (see Supporting Information S1). The pair of LBDs mediate the formation of a GRM1 dimer. Arrows indicate the direction of movement upon ligand binding that triggers activation of the receptor [25], [58]. The dashed box indicates an enlarged inset of the CRD shown in panel C. **C)**. Model of the GRM1 cysteine-rich domain (CRD). Wild-type and mutant side-chains at the nsSNP loci are shown in magenta and green stick format, respectively. Three disulphide bonds all conserved between GRM1 and GRM3 are shown in stick format and indicated by arrows.

Given that protein functions are often compartmentalized within specific domains [22], we examined if mutations clustered within a particular region of the gene. Although overall the hub genes contained no more nsSNPs than expected (0.6 nsSNPs per 100 codons) [23], multiple disease only nsSNPs clustered within a narrow stretch of sequence within particular genes. Ranking the genes using a measure of their cluster density and correcting for multiple testing (Supporting Information S1) showed that the most significant among these was a cluster found in *GRM1*, the metabotropic glutamate receptor subtype 1 (mGluR1) (Figure 1C). mGluR1 exists as a dimer at the post-synaptic membrane and upon ligand binding triggers a ‘Venus flytrap-like’ conformational change that is transduced by the cysteine-rich domain (CRD) to bring two membrane spanning G-protein couple receptor (GPCR) domains into apposition and activate a second messenger cascade [24] (Figure 2B). The *GRM1* cluster of 5 nsSNPs is highly unusual (permutation test, P = 0.004, see Supporting Information S1) spanning only 56 amino acid residues of the small CRD and neighbouring first transmembrane helix of the GPCR domain (Figure 2A). Moreover, sequence conservation analysis (Sift and PolyPhen, see Supporting Information S1) predicted that most of the deleterious nsSNPs in *GRM1* fell within this cluster (Figure 2A). All but one (K563N) of the nsSNPs found in the control cohort are excluded from the disease cluster (Figure 2A).

**Figure 3:**
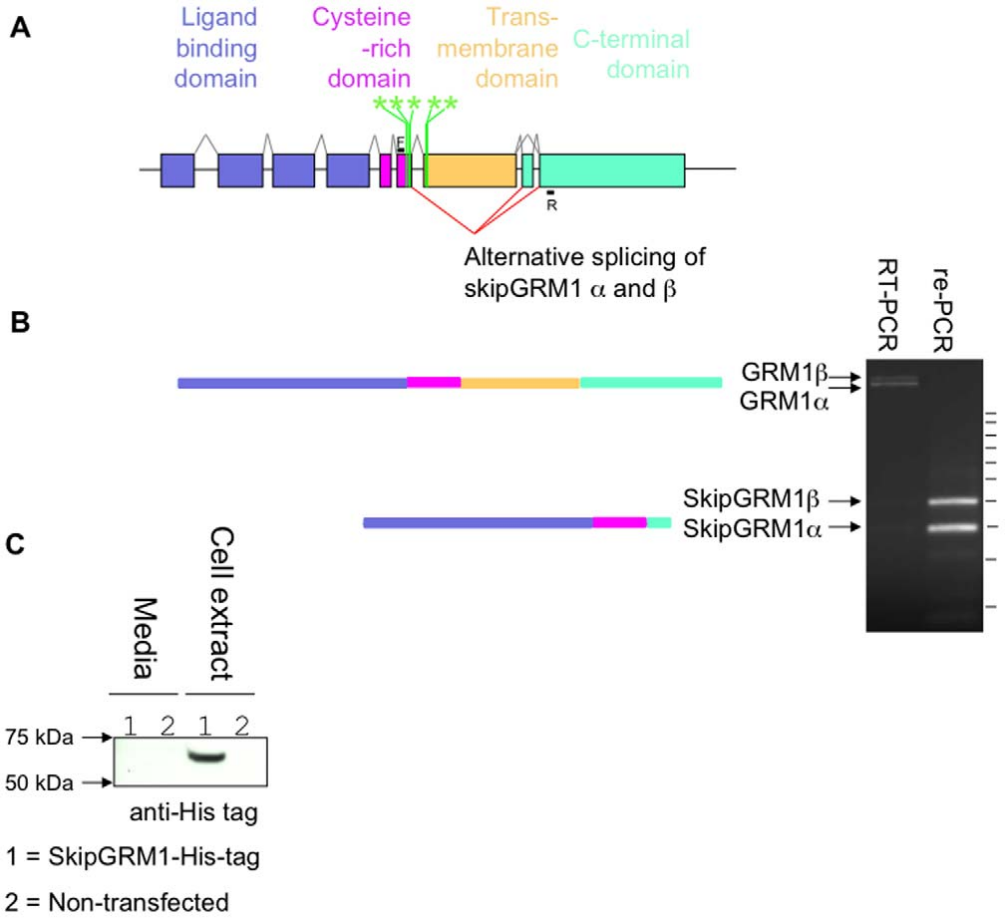
Mapping the *GRM1* nsSNP cluster onto the genomic structure of *GRM1.* **A)** Schematic showing the distribution of *GRM1* cluster nsSNPs within the gene structure of human *GRM1*. Canonical splicing of *GRM1* encoding the full-length protein is shown by grey lines connecting exons. A new alternative splice variant of *GRM1* is shown by red lines, in which the transmembrane GPCR domain of *GRM1* is skipped. Loci of *GRM1* SNP cluster are indicated in with vertical green bars. The predicted domain structure of skipGRM1 is shown. Exons are shown as rectangles. G RM1 is 410 kb long, but for clarity introns are not shown to scale. **B)** Detection of an exon-skipped GRM1 (skipGRM1α and β). Total RNA from human forebrain (sudden-death autopsy sample) was extracted. The first strand was generated by RT-PCR using poly-T oligonucleotides. Full length and skipGRM1 were detected using oligonucleotides specific to exon 6 (F; forward-primer) and exon 9 (R; reverse primer). 100 bp DNA ladder is indicated by horizontal black bars. Faint bands in the left lane corresponding to the novel splice junctions of skipGRM1α and β were gel-cleaned and further PCR amplified (right lane). The sequence of the PCR products encoding the splice junction between exon 6 and 8/9 were confirmed by DNA sequencing. The arrangements of protein domains for GRM1α and the skipGRM1α is shown. SkipGRM1α cDNA was cloned into a mammalian expression vector with a hexa-histidine tag (see Supporting Information S1). **C)** Growth media and lysate of His-tagged skipGRM1α transfected mammalian cells and non-transfected control samples were western blotted with anti-His tag antibody. SkipGRM1α expresses as a protein with an apparent molecular of approximately 68 kDa (see methods and the complete uncropped western blot image in Supporting Information S1).

In order to gain further insight into the potential effect of these polymorphisms on the GRM1 receptor, the nsSNP cluster was mapped onto available protein models (Figure 2A) and the gene structure (Figure 3A). Three of the nsSNPs were located in the CRD [25] for which a highly similar (46% identity) structural homolog is available (Figure 2B, C). The CRD is a small domain whose rigid structure is defined by seven disulphide bond-forming cysteines and is encoded by exons 5 and 6. These CRD nsSNPs lie in exon 6 and are within one to three residues of at least one cysteine. On nsSNP, L575V, is of particular interest since it encodes on an important β-turn with positive φ-torsion angle and is highly likely to form the nucleation site for protein folding [26], [27], which precedes the formation of disulphide bonds [28], [29]. The structural propensity for a valine in this position is negligible [30] (see Supporting Information S1). It is therefore possible these mutations affect protein folding [31], trafficking or activation of the receptor [32]. Preliminary data suggest that none of the mutations affect GRM1 signalling to phospholipase C, at least when transiently expressed in HEK293 cells (see Supporting Information S1). However, an effect of these mutations on alternate mGluR1 signalling pathways has not been excluded [33]. The CRD is present in all subtypes of the mGluR family and has been implicated in other inherited disease [32], which further supports the importance of this domain for the normal function of the receptor.

The two remaining nsSNPs of the cluster lie near the edge of exon 7, which encodes the transmembrane GPCR domain (Figure 2A), for which there is no suitable atomic protein model available. However, this cluster of 5 mutations together span the splicing boundary of exons 6 and 7 (Figure 3A) and bioinformatics analysis of splicing (see Supporting Information S1) predicts these mutations perturb sequence elements important for alternative splicing. Thus far no report of alternative splicing has been reported at this junction in *GRM1*. Therefore we examined if there was evidence of alternative splicing at this junction in human brain total RNA using cerebral cortex sudden-death autopsy samples. RT-PCR across the exon-exon boundary revealed the full length *GRM1* mRNA and the presence of an exon skipped RNA that encoded an mGluR1 completely lacking the GPCR domain and a short c-terminal domain (see Figure 3B and Supporting Information S1). The junction of this novel splice variant was sequenced, the cDNA was cloned, and the expected 68 kDa exon-skipped receptor (skipGRM1) protein was expressed in human cells (see Figure 3C and Supporting Information S1).

The proximity of the *GRM1* nsSNP cluster to the exon 6 donor site and exon 7 acceptor site suggest these nsSNPs might perturb the regulatory balance of alternative splicing of this receptor. Consistent with the discovery of *GRM1* nsSNP cluster is the discovery of exon skipping in a close homologue of *GRM1*, *GRM3* and its association with schizophrenia [34].

The total proportion of patients carrying one of these nsSNPs within this region of *GRM1* was 0.8%, whereas the proportion of controls was 0.2% (t-test, P = 0.015). This allele frequency in cases was similar to that of recurrent large deletions on chromosomes 22, 15 and 1, which were associated with schizophrenia at frequencies of 0.4%, 0.3%, and 0.3%, respectively [8]. Moreover, the repeated occurrence of apparently deleterious point mutations within this highly conserved region of *GRM1* suggest these mutations are ‘functionally recurrent’ and may be amenable to therapeutic intervention [35].

Several patients containing a mutation from the *GRM1* cluster also contained other nsSNPs distributed in the other hub genes, which prompted the question if individual variants alone were sufficient, or could act in concert with other molecular determinants. Such interactions have already been demonstrated by genetic studies in mice with compound mutations in MAGUK proteins [36] and NMDA receptor subunits [12] wherein the effect of carrying multiple compound mutations is greater than that of their parts, and results in more severe phenotypes. Similarly, since the ‘output’ of glutamate receptor activity is predicated on the coordinated action of the synaptic protein complex [20], [37], we asked if individual patients carrying multiple functionally important nsSNPs distributed within different components of the complex might contain a higher ‘genetic load’ contributing to disease.

The predicted deleterious effect of each nsSNP was measured taking into account multiple independent parameters including sequence conservation, effect on structural stability, proximity to phosphorylation sites, glycosylation sites and disulphide bonds (see Supporting Information S1). The mean deleteriousness score was not significantly different between nsSNPs found in the disease cohort and control. Individuals were then scored according to the sum of the predicted deleterious load contributed by their nsSNPs.

**Figure 4:**
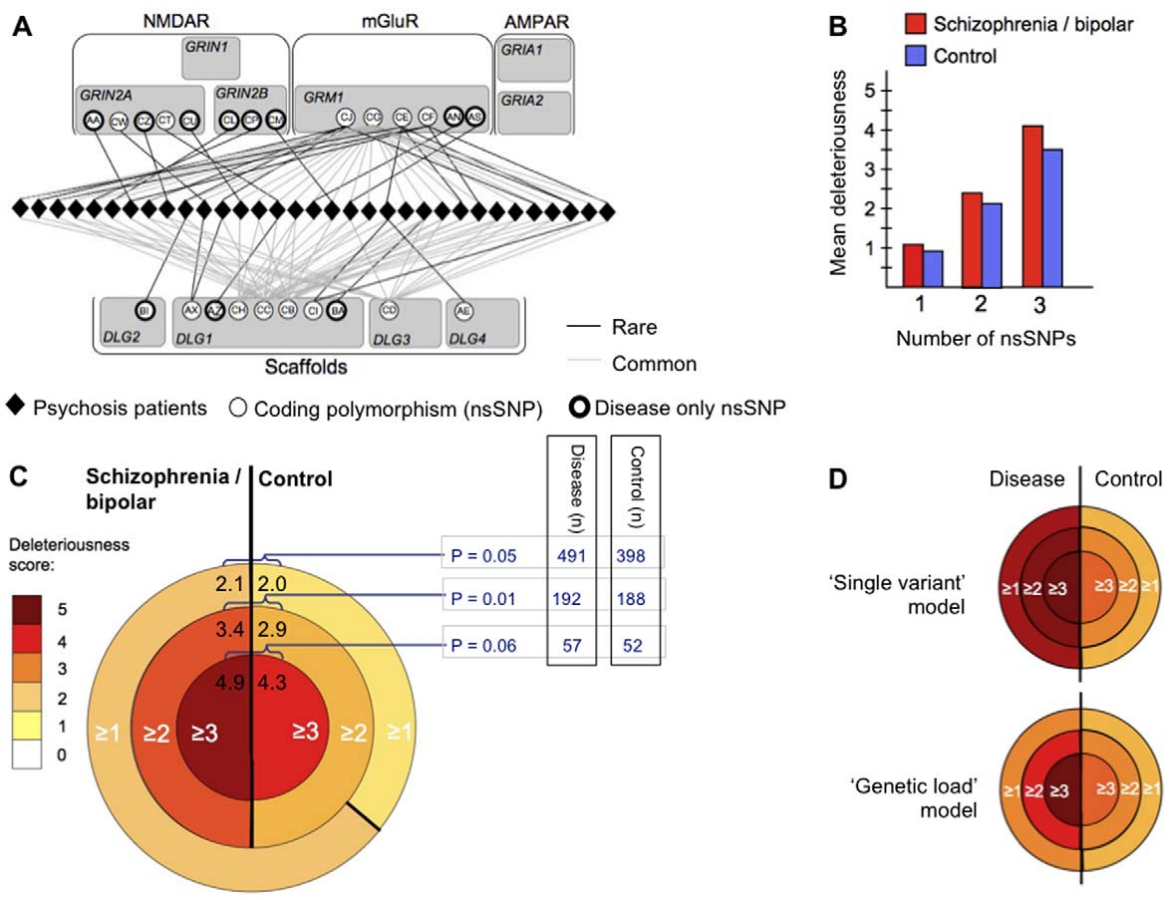
Analysis of the genetic load of multiple concurrent nsSNPs in individuals. **A)** Node diagram of concurrent nsSNPs from 33 schizophrenic and bipolar patients with ≥3 nsSNPs. Individuals are represented by rhombi that lie between the proteins (shaded grey). nsSNPs shown as circles are distributed among 6 proteins labelled with gene IDs (*GRIN2A*, *GRIN2B*, *GRM1*, *DLG1*, *DLG2*, *DLG3* and *DLG4*). Each unique nsSNP is labelled with a two-letter code (see Supporting Information S1 and datasheet S4 for the key). No nsSNPs were found concurrent with nsSNPs in *GRIN1*, *GRIA1* or *GRIA2* in any individuals in our cohorts. The combination of nsSNPs in any one patient is indicated by lines connecting an individual to multiple nsSNPs (black and grey lines are rare (<1%) and common variants (>1%), respectively). **B)** Bar graph showing the mean deleteriousness score per individual. This mean score was calculated for individuals (n = 509) with 1 nsSNP, 2 concurrent nsSNPs (n = 271) and 3 concurrent nsSNPs (n = 90) in disease and control (see Supporting Information S1). **C)** Concentric Venn diagram comparing subsets of schizophrenia/bipolar and control individuals with ≥1 nsSNPs (outer subset), ≥2 nsSNPs (middle subset) and ≥3 nsSNPs (inner subset). The average deleteriousness score (see supplementary datasheet S4) of each subset is labelled and shown as a heat-map. Significance of the difference between schizophrenia/bipolar and control was tested statistically by permutation protocols (see Supporting Information S1). The numbers of individuals from control and disease cohorts in each subset are shown as an inset table on the right. **D)** Hypothetical disease models of any one individual with disease. If a variant of any one allele in one individual is sufficient to cause disease, the following ‘single variant’ model can be represented with a concentric Venn diagram. This shows high deleteriousness within all subsets of individuals with varying numbers of concurrent nsSNPs. In contrast, if multiple concurrent variants must accumulate in an individual in order that the threshold of penetrance is reached, a ‘genetic load’ model can be represented in a concentric Venn diagram. This shows increasing deleteriousness over control that is proportional to the numbers of concurrent nsSNPs.

Combinations of two or more nsSNPs, referred to as concurrent nsSNPs (Figure 4A) were found in 380 individuals (192 schizophrenia/bipolar and 188 controls). When individuals with one nsSNP were compared to individuals with two and three concurrent combinations of nsSNPs, an increased load of deleteriousness in schizophrenic and bipolar disorder over controls was revealed (Figure 4B). The significance of this genetic load was estimated for subsets of individuals with increasing numbers of concurrent nsSNPs (Figure 4C). For individuals with at least one nsSNP there was a small 6% net increase in deleteriousness over control, (permutation, P = 0.05, see Figure 4C, *outermost subset of concentric Venn diagram*). In contrast, individuals with at least two concurrent nsSNPs the net increase in deleteriousness increased to 15%, (permutation, P = 0.01, Figure 4C, *second subset*). For three or more concurrent nsSNPs the net increase in deleteriousness was also 15%, but with a diminishing number of individuals in this subset significance is less (P = 0.06, Figure 4C *innermost subset*). Overall, these data are consistent with the concurrence of multiple nsSNPs having greater adverse effects in schizophrenia and bipolar disorder than in controls. Importantly, the genetic load detected here is distributed among components of a receptor-signaling complex that is fundamental to cognition and provides a plausible genetic revision of the long-standing glutamate hypothesis of psychosis disorders [2], [3].

## Discussion

*GRM1* identified in this association study has not previously been linked genetically with schizophrenia and bipolar disorder, although other evidence supports its involvement in these and other neurological disorders [38], [39], [40], [41]. *GRM1* has a well-established role in the commonest form of mental retardation, Fragile X syndrome [42]. The stabilisation of *DLG4* mRNA is mediated by the fragile X mental retardation protein (FMRP) and is enhanced by GRM1 activation [43]. In addition, FMRP regulates *DLG3* translation, and *DLG3* mutations also produce X-linked mental retardation [44]. Likewise an FMRP-associated gene, *CYFIP1*, containing a rare micro-deletion was found in a recent study of copy number variants in schizophrenia [9]. Thus, a pattern is emerging of functionally connected proteins [19] with deficits in different yet related psychiatric diseases that suggest *GRM1* and other genes encoding synaptic components are important for cognitive health generally [45], [46], [47]. The specific effect on synaptic function that this cluster of mutations interfere with remains to be identified, however, GRM1 plays an important role in synaptic scaling [48], a process that may be abrogated in schizophrenia [49] and perturbed by mutations in GRM1 that affect splicing.

Schizophrenia and bipolar have been described in psychiatry as a spectrum of many disorders with heterogeneous presentation, from which it has been proposed that genetics may lead to a sub-classification of these diseases into subtypes [50], [51]. Our data is consistent with a model in which disease subtypes might take the form of different nsSNP clusters such as that seen GRM1. In addition, the heterogeneity of particular combinations of concurrent rare and common nsSNPs and their increased genetic load of deleteriousness in schizophrenic and bipolar individuals may also contribute to the breadth of symptom profiles and drug responses.

Whilst categorizing disease subtypes is of immense importance, it is likely genome-wide studies that rely on the homogeneity of the disease cohort and measure the frequency distribution of tag-SNPs alone [52], [53], [54] are biased towards variation that is common to all subtypes of the disease and consequently may fail to detect the most penetrant, causal alleles specific to each disease subtype. Instead, the analysis of clustering and genetic load presented here identified putative causal alleles directly and their biological effects were assessed. Therefore we expect that expanding this multigenic approach using in-depth sequencing of other sets of genes, particularly other hubs within the synaptic proteome, will identify other nsSNP clusters, and genetic load that may define a range of neuropsychiatric diseases [19].

## Methods

### DNA samples and cohorts

The patient DNA samples (from blood) comprised Caucasian individuals contacted through the inpatient and outpatient services of hospitals in South East Scotland. A diagnosis of schizophrenia or bipolar disorder was based on information from an interview with the patient using the Schedule for Affective Disorders and Schizophrenia–Life time version (SADS-L) supplemented by case note review and frequently by information from medical staff, relatives and care givers. Final diagnoses, based on DSM-IV criteria (American Psychiatric Association 2000) were reached by consensus between two trained psychiatrists. The control group was composed of individuals that have undergone longitudinal assessment for cognitive ability and did not suffer from psychiatric disease [Lothian Birth Cohort 1921 (LBC 1921), Supporting Information S1]. The Multi-Centre Research Ethics Committee for Scotland approved the study and patients gave written informed consent for the collection of DNA samples for use in genetic studies. Re-sequencing and tag-SNP genotypes are shown in Datasheet S1 and S5, respectively. Relationships between individuals were investigated using the software GBR [55] with one individual removed from any pair showing relatedness. The results of the tag-SNP study are consistent with the absence of significant population stratification between cases and controls (test statistic inflation factor ‘lambda’ of 0.98, see Supporting Information S1).

### Exon re-sequencing

Exons and the flanking sequence of the ten candidate genes were extracted from the Vega database [56], which contains high-quality manually curated annotation. Primers were designed automatically using Primer3 to amplify the exon and at least 125 base pairs either side of the exon. Any exons failing automatic primer design had primers designed manually. Primer pairs were pre-screened to determine the optimum conditions for amplification. The majority of exons were amplified at 60°C. After amplification a sample of the products were visualised on an agarose gel, to confirm the size of the PCR product. The remaining PCR product was then ‘cleaned-up’ using two enzymes, Exonuclease 1 and Shrimp Alkaline Phosphatase. Bi-directional sequencing of amplicons was carried out using Big DyeTM chemistry. SNPs were called using ExoTrace, an algorithm developed for the detection of heterozygotes in sequence traces.

### Statistical analysis of nsSNP density

The density of nsSNPs was calculated as the number of nsSNPs within a genomic region divided by the length of that region (defined by the nsSNP positions on the outer edges of the cluster). The minimum cluster length was limited to 40 codons representing the approximate minimum size of a protein domain [57], since this avoids small clusters of a few closely spaced variants becoming highly ranked (see datasheet S3).

All possible groupings of nsSNPs within the 11 (10 hub proteins and one additional splice variant of DLG2) proteins were considered as a potential cluster. For each potential cluster, the significance of the deviation from the average density was tested using a Poisson model with an average 0.6 nsSNPs per 100 codons and the cluster with the most significant density increase was selected. It is of note that taking all the nsSNPs in our dataset, the average density (0.6 nsSNPs per 100 codons) is similar to that predicted for the human population (0.55 nsSNPs per 100 codons) [23].

The experiment-wide significance level of the best cluster within each gene was evaluated for each potential cluster by comparing its p-value from a Poisson distribution to the p-values from a Poisson distribution of randomly placed nsSNPs in each gene. This ‘extreme Poisson p-value distribution’ was constructed for each protein isoform in the following way:

1. 100,000 randomizations of positions of affected residues were performed.
2. In each randomization replicate, all contiguous subsets of nsSNPs were compared to the mean SNP density in that protein isoform and the Poisson p-value of the densest subset was recorded for each replicate.
3. One hundred thousand randomizations on each isoform allowed us to construct a distribution of extreme Poisson p-values for each protein isoform.

Finally, each protein's most dense cluster Poisson p-value was compared to the distribution of Poisson P-values obtained by randomization. The computed significance of each protein's best cluster was corrected using the Benjamini-Hochberg false discovery rate.

### Statistical analysis of predicted genetic load of deleteriousness

Multiple parameters (see Supporting Information S1) were used to score the predicted effect of each nsSNP (scores for each parameter are presented in datasheets S2 and S4). The significance of the difference in the average deleteriousness scores for individuals in the case and controls groups was assessed by permutation. The case and control labels were randomly permuted 10,000 times to obtain the distribution of the expected difference in average deleteriousness under the null hypothesis. As it would be expected that the disease group had an increased deleteriousness on average, the alternative hypothesis is one-sided and its significance was evaluated by calculating the proportion of permuted test statistics (datasheet S9, *column J*) greater than or equal to the observed value (datasheet S9, *column I*). These permutation tests were also performed on restricted subsets of individuals who carried at least one, two or more and three or more non-synonymous variants. Restricting the sample further into groups with four or more was not investigated due to the restricted sample sizes that would result in low statistical power to significantly detect any difference. P-values are shown in supplementary datasheet S9, *column L*.

### Detection and cloning of exon-skipped GRM1

Total RNA was extracted from sudden-death autopsy brain samples (dorso-lateral prefrontal cortex). GRM1 splice variants were amplified, sequenced and cloned by PCR (see Figure 4 and Supporting Information S1).

### Tag-SNP genotyping

Tag-SNPs within a wider set of 169 MASC genes and 96 other candidates (see datasheets S6 and S7) were genotyped. tag-SNPs were selected for 265 genes using the Tagger Pair-wise method on HapMap PhaseII CEU data. We aimed to assay 10 kb flanks upstream and downstream of each gene and used the parameters minimum R^2^ of 0.8 and minimum minor allele frequency of 0.05. SNPs were submitted to Illumina for GoldenGate assay design and those judged to be viable assay targets (design score > = 0.4) were ordered as 3 Oligo Pool Assays. 12 assays were repeated in all 3 pools as a quality control measure. Consistent with other genome wide association studies of schizophrenia and bipolar disorder [10], [21], tag-SNP genotyping was unable to find any single common variants significantly associated with either schizophrenia or bipolar disorder. However, these data provided evidence that the ten genes selected for sequencing showed significantly more association with disease than would be expected by chance (rank sum P = 0.0034, see Supporting Information S1 and Datasheet S8). Overall, this tag-SNP data revealed only marginal association and is incapable of directly identifying the specific mutations that contribute to disease. Detailed methods have been described in Supporting Information S1.

## Supporting Information

Supporting Information S1 Document contains detailed methods for the detection and analysis of coding variants, investigations into the GRM1 nsSNP cluster, tag-SNPs, and the control cohort (LBC).
(DOC)

Datasheet S1 Exon re-sequencing genotypes from schizophrenia, bipolar disorder and control (LBC). First and second columns indicate patient code and their cohort, respectively. The remaining columns are headed by nsSNP names, under which are the genotypes for each individual.
(XLS)

Datasheet S2 nsSNP name, chromosomal locus, frequency in each cohort, functional annotation, and scoring for each nsSNP. All nsSNPs have also been deposited in G2Cdb^1^ and dbSNP^2^.
(XLS)

Datasheet S3 details of the statistics for identifying nsSNP clusters and estimation of their significance by randomization analysis.
(XLS)

Datasheet S4 Identities and scores of individuals with concurrent nsSNPs.
(XLS)

Datasheet S5 tag-SNP genotypes of schizophrenia and LBC individuals.
(TXT)

Datasheet S6 List of genes tagged for genotyping with permutation test p-value for each gene and rank.
(XLS)

Datasheet S7 Fisher's exact test of association for each tag-SNP.
(XLS)

Datasheet S8 Table 1: List of protein-protein interactions used in linear regression analysis (see section 3.8 of the supplementary information). Table 2: Lists for each gene the number (calculated using Table 1) of interactions the protein (encoded by the gene) makes with the 10 hub proteins.
(XLS)

Datasheet S9 Statistics for concurrent nsSNPs analysis showing an increasing net deleteriousness correlated with increasing nsSNP count.
(XLS)
