# Supplementary material for: Clustered Coding Variants in the Glutamate Receptor Complexes of Individuals with Schizophrenia and Bipolar Disorder
Source: PLoS One. 2011 Apr 29;6(4):e19011. doi: 10.1371/journal.pone.0019011 (PMC3084736; doi:10.1371/journal.pone.0019011)
Supplement: Supporting Information S1 — Document contains detailed methods for the detection and analysis of coding variants, investigations into the GRM1 nsSNP cluster, tag-SNPs, and the control cohort (LBC). (DOC) [file pone.0019011.s001.doc]

**Supporting Information S1.**

**1. RAW DATA**

Data are contained in seven Datasheets:

**Datasheet_S1_nsSNPgenetypes.xls**: Exon re-sequencing genotypes from schizophrenia, bipolar disorder and control (LBC). First and second columns indicate patient code and their cohort, respectively. The remaining columns are headed by nsSNP names, under which are the genotypes for each individual.

**Datasheet_S2_nsSNPs.xls**: nsSNP name, chromosomal locus, frequency in each cohort, functional annotation, and scoring for each nsSNP. All nsSNPs have also been deposited in G2Cdb^1^ and dbSNP^2^.

**Datasheet_S3_clusteranalys.xls**: Shows details of the statistics for identifying nsSNP clusters and estimation of their significance by randomization analysis. See supplementary information section 2.5.

**Datasheet_S4_concurrentnsSNPanalysis.xls**: Identities and scores of individuals with concurrent nsSNPs. See supplementary information section 2.6 and 2.7.

**Datasheet_S5_tagSNPgenotypes.txt**: tag-SNP genotypes of schizophrenia and LBC individuals. See supplementary information section 3.2

**Datasheet_S6_tagSNPgenes.xls**: List of genes tagged for genotyping with permutation test p-value for each gene and rank. See supplementary information section 3.5.

**Datasheet_S7_tagSNPpvalues.xls:** Fisher’s exact test of association for each tag-SNP. See supplementary information section 3.4.

**Datasheet_S8_tagSNP_networkanalysis.xls**: Table 1: List of protein-protein interactions used in linear regression analysis (see section 3.8 of the supplementary information). Table 2: Lists for each gene the number (calculated using Table 1) of interactions the protein (encoded by the gene) makes with the 10 hub proteins. See supplementary information section 3.8

**Datasheet_S9_concurrent_nsSNP_permuation.xls**: Statistics for concurrent nsSNPs analysis showing an increasing net deleteriousness correlated with increasing nsSNP count. See supporting information section 2.7.

**2. METHODS FOR RARE VARIANTS**

**2.1 DNA samples, cohorts and data**

The patient samples comprised Caucasian individuals contacted through the inpatient and outpatient services of hospitals in South East Scotland. A diagnosis of schizophrenia or bipolar disorder was based on information from an interview with the patient using the Schedule for Affective Disorders and Schizophrenia–Life time version (SADS-L) supplemented by case note review and frequently by information from medical staff, relatives and care givers. Final diagnoses, based on DSM-IV criteria [American Psychiatric Association 2000] were reached by consensus between two trained psychiatrists. The Multi-Centre Research Ethics Committee for Scotland approved the study and patients gave written informed consent for the collection of DNA samples for use in genetic studies.

Control dataset: Ascertainment and recruitment of the Lothian Birth Cohort of 1921 samples are described in more detail elsewhere^3^.

**2.2 Hub genes targeted for exon re-sequencing**

The identities of synaptic proteins that bind the NMDA receptor have been determined and include interactions with membrane associated guanylate kinases (MAGUKs)^4^. AMPA receptors also anchor MAGUKS at the synapse^5^. Together these receptors and MAGUKs assemble with many other synaptic proteins that from MAGUK-associated signalling complexes (MASC)^6^. Consequently, the 10 hub genes targeted for re-sequencing included key members of the glutamate receptor families: NMDA receptor subunits (GRIN1, GRIN2A, GRIN2B), AMPA subunits (GRIA1, GRIA2) and mGluR (GRM1) in addition to the paralogous MAGUK interactors (DLG1, 2, 3 and 4).

**2.3 Exon re-sequencing method**

Exons and the flanking sequence of the ten candidate genes were extracted from the Vega database^7^, which contains high-quality manually curated annotation. Primers were designed automatically using Primer3 to amplify the exon and at least 125 base pairs either side of the exon. Any exons failing automatic primer design had primers designed manually. Primer pairs were pre-screened to determine the optimum conditions for amplification. The majority of exons were amplified at 60°C. After amplification a sample of the products were visualised on an agarose gel, to confirm the size of the PCR product. The remaining PCR product was then ‘cleaned-up’ using two enzymes, Exonuclease 1 and Shrimp Alkaline Phosphatase. Bi-directional sequencing of amplicons was carried out using Big DyeTM chemistry. SNPs were called using ExoTrace, an algorithm developed for the detection of heterozygotes in sequence traces.

nsSNPs were mapped to exon loci in ensembl^8^ (see datasheet S2). The longest protein transcripts including the nsSNP locus were mapped to uniprot^9^. All nsSNPs were mapped to the primary splice variant of each gene with the exception of three nsSNPs (see supporting datasheet S2; NT_033927.7_14250750, NT_033927.7_14250900, and NT_010718.15_6708779), which were found in an exon only present in an alternative splice form of DLG2. One nsSNP (NT_010718.15_6708779) was found within a hypothetical exon in DLG4, for which no evidence of this exon’s expression in a protein was available. Consequently, this nsSNP was excluded from all analysis of protein features. Uniprot sequences were used for all subsequent analyses of proteins encoding the 10 hub genes.

**2.4 Frequency analysis**

nsSNP frequency is shown for each cohort in datasheet S2. Frequencies ranged from less than 0.1 to 45%. Eight of the 63 nsSNPs had a frequency greater than >1% and are labeled as ‘common’ in the main text and figures. The remaining 55 nsSNPs are referred to as ‘rare’. No single nsSNP showed significant association (Fisher’s exact test) with either disease cohorts (see datasheet S2, *column R*).

**2.5 Cluster analysis of disease nsSNPs**

Since proteins are organized into structural and functional domains, we asked if different rare nsSNPs from different individuals clustered within a narrow defined region of the same protein (Figure S2A,B,C). Plotting nsSNPs along the length of each protein shows the distribution of nsSNPs and suggests there is a striking cluster of disease-associated nsSNPs in GRM1 (Figure S1).

A method was designed that achieves the following:

1. Automatically identifies the densest cluster of nsSNPs in each protein isoform testing all possible ‘window sizes’.
2. Tests the significance of each cluster by randomization.

The density of nsSNPs was calculated as the number of nsSNPs within a genomic region divided by the length of that region (defined by the nsSNP positions on the outer edges of the cluster). The minimum cluster length was limited to 40 codons representing the approximate minimum size of a protein domain^10^, since this avoids small clusters of a few closely spaced variants becoming highly ranked.

All possible groupings of nsSNP with the 11 (10 hub proteins and one additional splice variant of DLG2) proteins were considered as a potential cluster. For each potential cluster, the significance of the deviation from the average density was tested using a Poisson model with an average 0.6 nsSNPs per 100 codons and the cluster with the most significant density increase was selected. It is of note that taking all the nsSNPs in our dataset, the average density (0.6 nsSNPs per 100 codons) is similar to that predicted for the human population (0.55 nsSNPs per 100 codons; see datasheet S3)^11^.

The experiment-wide significance level of the best cluster within each gene was evaluated for each potential cluster by comparing its p-value from a Poisson distribution to the p-values from a Poisson distribution of randomly placed nsSNPs in each gene (see Figure S1). This ‘extreme Poisson p-value distribution’ was constructed for each protein isoform in the following way:

1. 10,0000 randomizations of positions of affected residues were performed.
2. In each randomization replicate, all contiguous subsets of nsSNPs were compared to the mean SNP density in that protein isoform and the Poisson p-value of the densest subset was recorded for each replicate.
3. One hundred thousand randomizations on each isoform allowed us to construct a distribution of extreme Poisson p-values for each protein isoform.

Finally, each protein's most dense cluster Poisson p-value was compared to the distribution of Poisson P-values obtained by randomization (see Figure S1). The computed significance of each protein's best cluster was then corrected using the Benjamini-Hochberg false discovery rate.


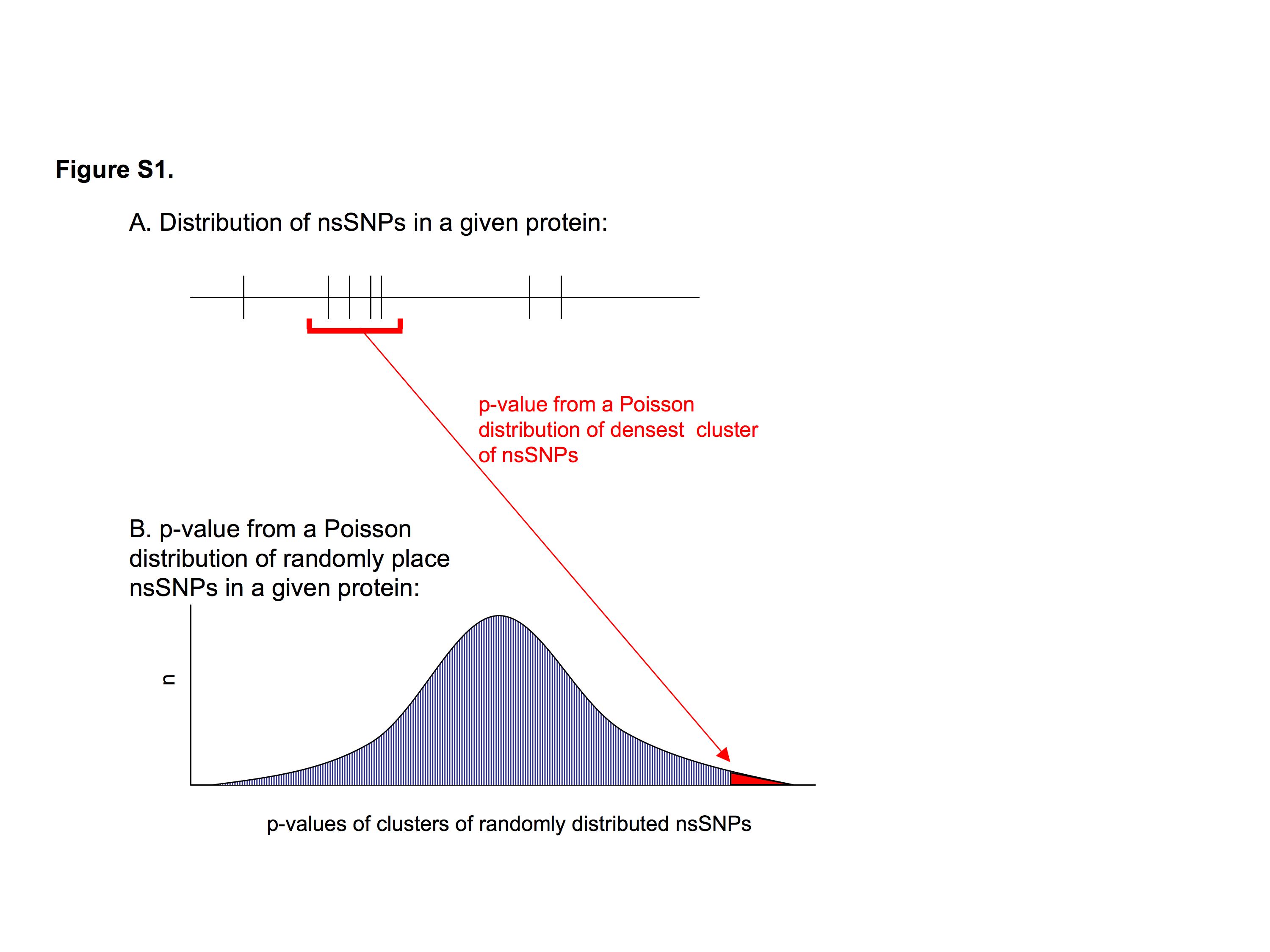


Clustering tests was performed on both nsSNP that were exclusive to the disease cohorts (Figure S2A) and nsSNPs found at least once in a disease cohort (Figure S2B). The higher stringency computation identified several non-overlapping nsSNP clusters (indicated with green brackets in Figure S2) and was used to rank the genes (Figure 1B in main text).


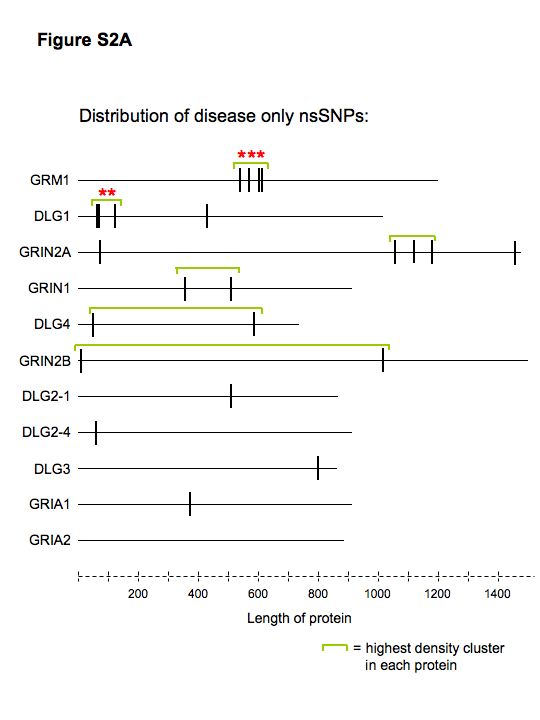

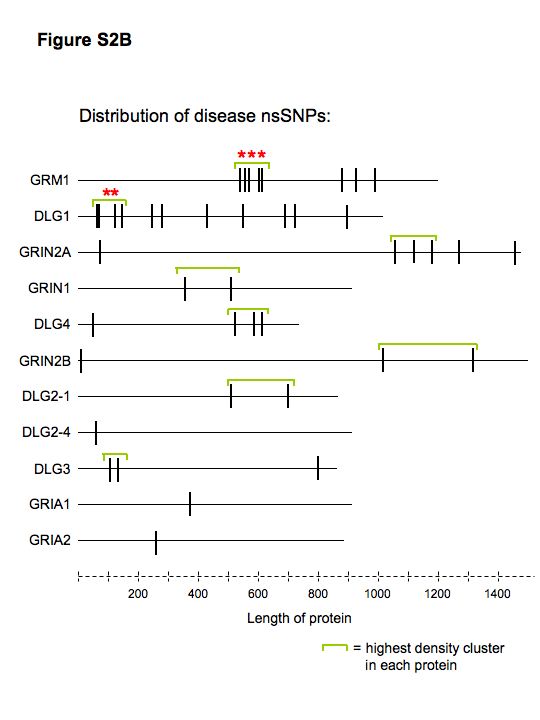

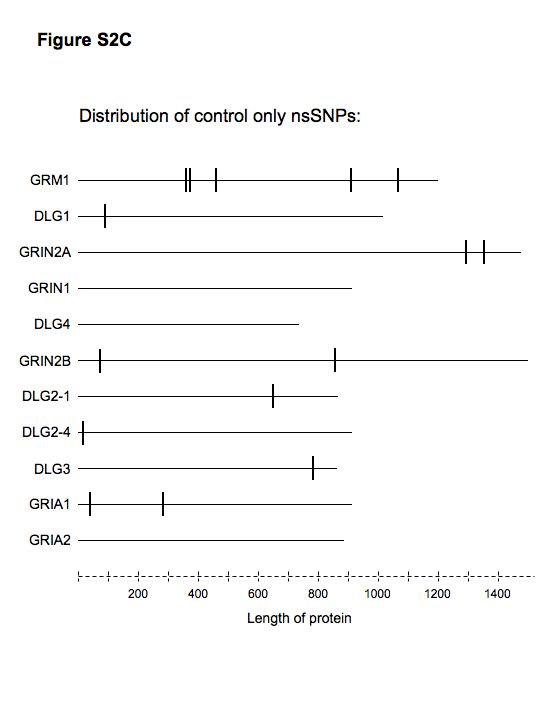


Following automated nsSNP cluster ranking, the most significant cluster, which came from GRM1, was manually assessed. The predicted deleteriousness of the nsSNPs in GRM1 (see Figure 1B in main text) was evaluated by polyphen^12^ and sift^13^ (see datasheet S2).

A highly homologous structural model spanning as many of the nsSNPs within the cluster as possible was found using fugue^14^. Side-chains were modelled using scwrl^15^. It was noted that one of the nsSNPs within the cluster was positioned at a type I' β turn, in which the nsSNP had a positive φ torsion angle. It was apparent that this backbone torsion angle is unfavourable for the disease variant in which valine occupies this position (L575V) because the beta-branched side-chain of valine would eclipse the main-chain. We used SPASM^16^ to search the protein databank for type I' β turns with a leucine or a valine in this conformation. 160 non-redundant structures contain leucine at this position, whereas only one non-redundant structure was found with a valine at this position. It is therefore likely that the native backbone conformation would not tolerate a valine.

**2.6 Deleteriousness score**

Multiple parameters were used to score the predicted effect of each nsSNP (scores for each parameter are presented in datasheet S2).

*Conservation***.** The probability of the predicted effect of a nsSNP on a protein was assessed using polyphen^12^ and sift^13^ (see datasheet S2). Both algorithms search for homologues (<90% identity) to determine the conservation of the site in which the nsSNP occurs. Each algorithm gives a prediction above a default threshold over which the nsSNP is predicted damaging. There was not complete overlap in the predictions by these algorithms thus both were employed. All nsSNPs returned a result except for two nsSNPs (NT_010393.15_1170125 and NT_033927.7_14250900) located at the far N-terminus of the protein, for which insufficient homologues could be found to obtain a sift score (see datasheet S2).

*Structural effects***.** High confidence (z-score >8) structural homologues spanning regions containing the nsSNP were found using fugue^14^. Side-chains were modelled on fugue-generated backbone models using scwrl3^15^. Structural models of the nsSNP variant structures were generated by andante^17^. A measure of structural deleteriousness was calculated in sdm^18^ by comparing the two structures and the change in protein stability (pseudo ΔΔG; see datasheet S2).

*Functional effects***.** A measure of functional deleteriousness was estimated by identifying nsSNP loci that overlap or are in the proximity (+/- 3 residues) of glycosylation, ligand-binding sites, phosphorylation sites and disulphide-bonded cysteines. Predicted glycosylation sites on extracellular regions of proteins and ligand-binding sites were taken from NetGly and NetLig, respectively. Empirically identified phosphorylation-sites were obtained from scansite^19^. Predicted phosphorylation sites were retrieved from netphos^20^. Predicted sites for extracellularly located regions of the protein were excluded from the analysis to minimize miscalling phosphorylation sites. Disulphide bonds were identified for proteins for which a structure or structural homologue was available.

*Scoring*. Each nsSNP was scored in all the parameters used for predicting deleteriousness, namely sequence conservation, structural stability and functional effects (Figure S3A). Each of these analyses was independent of the other. Accordingly, scores from each parameter were summed giving a total score attributed to each nsSNP (see datasheet S2). We identified individuals with multiple nsSNPs (see datasheet S1). To calculate the total deleterious load of each individual we summed the deleteriousness scores of their constituent nsSNPs (see Figure S3B). Patient codes, concurrent nsSNPs and their score are listed in datasheet S4.


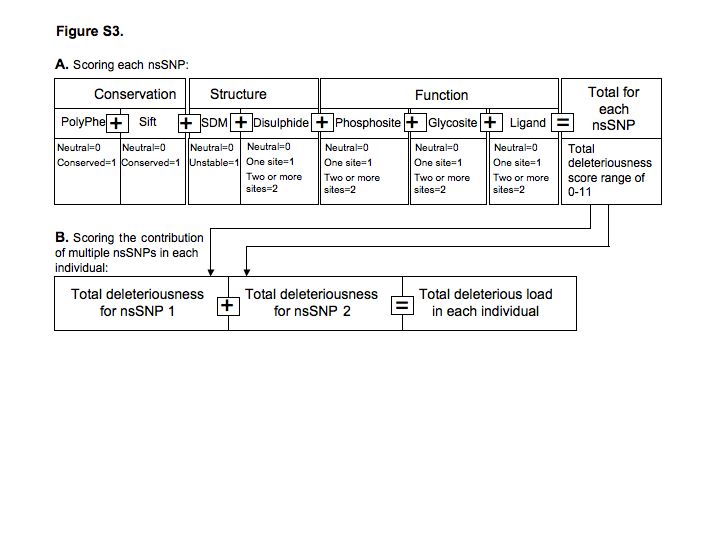


**2.7 Association and significance testing of concurrent nsSNPs**

The significance of the difference in the average deleteriousness scores for individuals in the case and controls groups was assessed by permutation. The case and control labels were randomly permuted 10000 times to obtain the distribution of the expected difference in average deleteriousness under the null hypothesis. As it would be expected that the disease group had an increased deleteriousness on average, the alternative hypothesis is one-sided and its significance was evaluated by calculating the proportion of permuted test statistics (supporting datasheet S9, *column J*) greater than or equal to the observed value (supporting datasheet S9, *column I*).

Similar permutation tests were performed on restricted subsets of individuals who carried at least one, two or more and three or more non-synonymous variants. Restricting the sample further into groups with four or more was not investigated due to the restricted sample sizes that would result in low statistical power to significantly detect any difference. It is of note that the average difference in deleteriousness per individual between cases and controls increased monotonically with increasing number of nsSNPs (see main text Figure 3B and 3C and supporting datasheet S9, *column I*) consistent with increased genetic load of deleteriousness for those with the disease. P-values are shown in supporting datasheet S9, *column L*.

**2.8 Predicted effect of GRM1 cluster nsSNPs on splicing**

The regulatory elements that mediate constitutive and alternative splicing in particular, are at present ill defined. However, there is evidence that exonic elements play a part in these RNA regulatory events and may contain mutations responsible for disease^21^. We scored the potential for nsSNPs found in the GRM1 cluster to cause splicing using the web-tool skippy (http://research.nhgri.nih.gov/skippy/input.shtml)^22^. Four of the five nsSNPs in the GRM1 cluster are predicted to interfere with features that modulate splicing.

**3.0 Empirical investigation of GRM1 clusters SNPs**

**3.1 Detecting GRM1 splice variants in human forebrain autopsy samples**

Human sudden-death autopsy samples from the dorsolateral prefrontal cortex (Brodmann’s area 8/9) were a generous gift from Professor Colin Smith, MRC Brain Bank, University of Edinburgh). Total RNA was extracted from 129 mg tissue using 0.5 ml QIAZol reagent and a pellet pestle motor (Kontes). The homogenate was diluted to a final volume of 5 ml with QIAzol before total RNA was prepared using the RNeasy lipid tissue midi kit (Qiagen). Samples were eluted in 150 μl RNase free water and stored at -80^o^C.

RNA was reverse transcribed to produce the first strand of cDNA in a total volume of 40 μl. 20 ul human forebrain total RNA, 2 μl 500 ng/µl oligo dT (Invitrogen), 2 μl 10 mM dNTPs (Invitrogen) were incubated at 65^o^C for 7 min then transferred to ice, to which 8 μl 5x Superscript II buffer (Invitrogen), 4 μl 100 mM DTT, and 2 µl water were added. The sample was incubated at 42^o^C for 2 min, after which 2 μl superscript II reverse transcriptase (Invitrogen) was added. The sample was then incubated on a thermocycler at 42^o^C for 50 min, followed by 70^o^C for 15 min. First strand cDNA samples were stored at -20^o^C.

First strand cDNA samples were used for detecting GRM1 splice isoforms and cloning the exon-skipped GRM1a cDNA.

A forward primer complementary to GRM1 exon 6 (labelled ‘F’ in Figure 3: cggaaaggagaagtgagctg) and a reverse primer complementary to GRM1 exon 9 (labelled ‘R’ in Figure 3: gccgtctcattggtcttcac) were used to detect splice variants of GRM1 by PCR. PCR was performed using: 2 μl first strand cDNA, 0.5 μl dNTPs, 2.5 μl 10x Hotstar buffer (Qiagen), 1 μl 10 μM primer F, 1 μl 10 μM primer R, 17.85 μl miliQ water, 0.15 μl *taq* Hotstar (Qiagen). Samples were incubated on a thermocycler: 1) 95^o^C 15 min, 2) 95^o^C 15 s, 3) 55^o^C 1 min, 4) 68^o^C 1 min, 5) Repeat 34x steps 2-4, 6) 68^o^C 10 min. Samples were analysed by 2% agarose gel electrophoresis.

Faint bands were excised and gel cleaned (Promega), from which gel band DNA samples were eluted in 16 μl. Gel band DNA samples were used for further amplification by PCR. Gel bands were ligated into a pDRIVE vector (Qiagen) and sequenced to confirm the novel splice junction of exon-skipped GRM1 (see Figure 3B).

**3.2 Cloning human secreted GRM1a cDNA**

The exon-skipped GRM1α cDNA with a predicted length of 1821 bp:

atggtcgggctccttttgttttttttcccagcgatctttttggaggtgtc

ccttctccccagaagccccggcaggaaagtgttgctggcaggagcgtcgt

ctcagcgctcggtggccagaatggacggagatgtcatcattggagccctc

ttctcagtccatcaccagcctccggccgagaaagtgcccgagaggaagtg

tggggagatcagggagcagtatggcatccagagggtggaggccatgttcc

acacgttggataagatcaacgcggacccggtcctcctgcccaacatcacc

ctgggcagtgagatccgggactcctgctggcactcttccgtggctctgga

acagagcattgagttcattagggactctctgatttccattcgagatgaga

aggatgggatcaaccggtgtctgcctgacggccagtccctccccccaggc

aggactaagaagcccattgcgggagtgatcggtcccggctccagctctgt

agccattcaagtgcagaacctgctccagctcttcgacatcccccagatcg

cttattcagccacaagcatcgacctgagtgacaaaactttgtacaaatac

ttcctgagggttgtcccttctgacactttgcaggcaagggccatgcttga

catagtcaaacgttacaattggacctatgtctctgcagtccacacggaag

ggaattatggggagagcggaatggacgctttcaaagagctggctgcccag

gaaggcctctgtatcgcccattctgacaaaatctacagcaacgctgggga

gaagagctttgaccgactcttgcgcaaactccgagagaggcttcccaagg

ctagagtggtggtctgcttctgtgaaggcatgacagtgcgaggactcctg

agcgccatgcggcgccttggcgtcgtgggcgagttctcactcattggaag

tgatggatgggcagacagagatgaagtcattgaaggttatgaggtggaag

ccaacgggggaatcacgataaagctgcagtctccagaggtcaggtcattt

gatgattatttcctgaaactgaggctggacactaacacgaggaatccctg

gttccctgagttctggcaacatcggttccagtgccgccttccaggacacc

ttctggaaaatcccaactttaaacgaatctgcacaggcaatgaaagctta

gaagaaaactatgtccaggacagtaagatggggtttgtcatcaatgccat

ctatgccatggcacatgggctgcagaacatgcaccatgccctctgccctg

gccacgtgggcctctgcgatgccatgaagcccatcgacggcagcaagctg

ctggacttcctcatcaagtcctcattcattggagtatctggagaggaggt

gtggtttgatgagaaaggagacgctcctggaaggtatgatatcatgaatc

tgcagtacactgaagctaatcgctatgactatgtgcacgttggaacctgg

catgaaggagtgctgaacattgatgattacaaaatccagatgaacaagag

tggagtggtgcggtctgtgtgcagtgagccttgcttaaagggccagatta

aggttatacggaaaggagaagtgagctgctgctggat**t**tgcacggcctgc

aaagagaatgaatatgtgcaagatgagttcacctgcaaagcttgtgactt

gggatggtggcccaatgcagatctaacagttctaatggcaagtctgtgtc

atggtctgaaccaggtggaggacaggtgcccaagggacagcatatgtggc

accgcctctctgtgcacgtga

Nucleotides 1-955 and 933-1821 were amplified in two separate PCR reactions with primers F1/ R1 (CCGGAATTCGCCACCatggtcgggctccttttgt and catcacttccaatgagtgagaac) and F2/R2 (gttctcactcattggaagtgatg and TTTTTTCCTTGCGGCCGCgccgtctcattggtcttcac), respectively, using the PCR conditions describecd in section 3.1. Each PCR product was gel cleaned and ligated into a pDRIVE vector using the PCR cloning kit (Qiagen) to produce clones TA-1 and TA-2. Clones of TA-1 and TA-2 lacking PCR errors (screened by sequencing) were used as templates for nested PCR assembly of the cDNA using a proof-reading polymerase, *pfx* (Invitrogen) and the primers above. PCR products were gel-cleaned between each PCR reaction and the assembled cDNA was ligated into pLEXm^23^ and pCDNA3.1 (Invitrogen) using restriction enzymes (EcoRI/NotI and EcoRI/BamHI, respectively. A His-tagged cDNA clone of pLEXm skipGRM1α was also generated by inserting nucleotides encoding 6 histidines downstream of skipGRM1 and upstream of the stop codon. All cDNAs encoded the expected sequence of the skipGRM1α, except for the presence of a synonymous T→A polymorphism at position 1638 (position shown in red in sequence above).

**3.3 Heterologous expression of exon-skipped GRM1a**

10^5^ human embryonic kidney (HEK193T) cells were passaged into 3 ml fresh media (DMEM, 10% FBS, glutamine, pen/strep) and cultured in 6-well plates at 37^o^C, 5% CO_2_ for three days. Before transfection, cells were washed with DMEM only and the media was exchanged with 2.5 ml OptiMEM. 2.5 μg pLEXm skipGRM1α-his tag vector in 250 μl OptMEM was added to 3.75 μg polyethylenimine in 250 μl OptiMEM, vortexed for 10 s and incubated at room temperature for 10 m before adding to cells. 2.5 μg pmacGFP was transfected similarly in a control culture. Following 3 days expression at 37^o^C, 5% CO_2_, the media was aspirated and cells were lysed with 125μl 1% triton X-100, 0.5% Na deoxycholate, 0.1% SDS, 50 mM tris.Cl pH8 for 1 h at 4^o^C. 30 μl media and cell extract were mixed with SDS loading buffer, 100 mM DTT, briefly sonicated and separated by 4-12% bis-tris SDS-PAGE. After protein transfer to a PVDF membrane, the membrane was blocked with 1.25% casein, 1.25% BSA, TBS, 0.1% triton X-100 for 1 h at room temperature and incubated with 1:5000 rabbit polyclonal anti-Histag-HRP conjugate (Abcam ab1187) in 0.5% casein, 1.25% BSA, TBS, 0.1% triton X-100 for 1 h at room temperature. The membrane was washed 5x 5 min with TBS, 0.1% triton X-100 and briefly rinsed with PBS. Immunostained protein bands were detected by chemiluminescence (ECL advance, GE healthcare) and exposure to film (see Figure 3C in the main text and supporting figure S4, *left*). To control for protein loading, the membrane was stripped for 45 min with 1X reblot plus mild (Millipore), blocked, incubated with 1:1500 mouse anti-pan actin (Abcam ab3280), washed, incubated with 1:20,000 anti-mouse antibody HRP conjugate (Millipore), washed and detected as before (see supporting figure S4B, *right*). Approximately equal amounts of actin were detected in the lysate of skipGRM1α transfected and control cells.


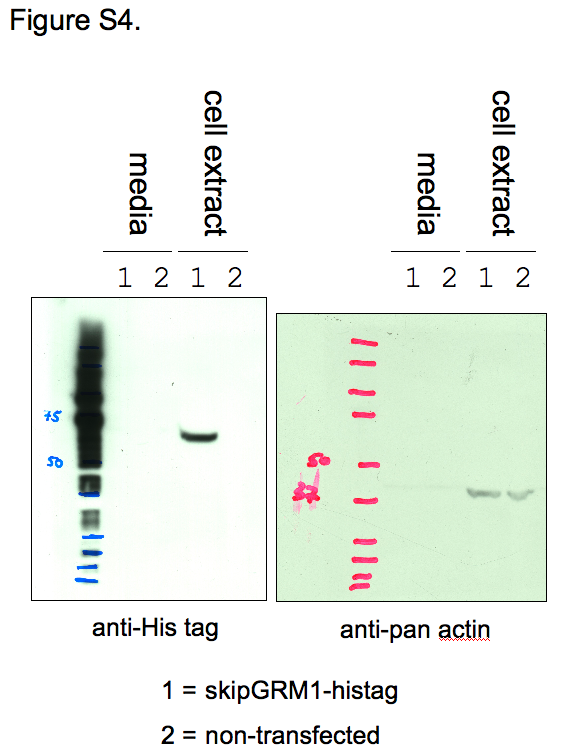


**3.4 Activity assay of recombinant GRM1 mutants**

A plasmid vector (pCDNA3.1) carrying the cDNA encoding human mGluR1a was a generous gift from Dr. Menelas Pangalos (Pfizer Incorporated, New York). 5 different mutants were generated each corresponding to one of the nsSNPs found in the GRM1 nsSNP cluster (T548M, K563N, L575V, L602M, I604M). Mutations were generated using primers encoding each mutation and the QuikChange mutagenesis kit (Qiagen). Putative mutants were confirmed by DNA sequencing the clones.

Activity assays were performed in cell culture. Briefly, confluent HEK293 cells were passaged [1:7 cells in culture medium (DMEM, GlutaMAX I, 10% fetal calf serum, +penicillin/streptomycin (Invitrogen)] into T25 flasks (coated in poly-D-lysine) and cultured for 32 h (37^o^C, 5% CO_2_ humidified atmosphere). Cells were briefly washed with OptiMEM (Invitrogen) before 4 mL OptiMEM and the transfection mix [12.5 μL 400 ng/μL plasmid, 5 μL lipofectamine2000 (Invitrogen), 1 mL OptiMEM prepared 20 min before] were added to the cells and cultured overnight. Transfection was terminated by replacing with fresh culture medium for 6 h before cells were passaged into 24-well plates at a density of 2.5x10^5^ cells/well in culture medium supplemented with 2.5 μCi/mL *myo*-[^3^H]-inositol and cultured for a further 48 h.

Glutamic-pyruvic transaminase (3 U/mL) and pyruvate (5 mM) were added before stimulations to minimize free glutamate concentration. LiCl was added for 30 min before agonist addition. The indicated concentrations of quisqualate (0.003-30 μM) were added and incubations terminated after 20 min by aspiration and addition of ice-cold 0.5 M TCA. Cells were neutralized and the [^3^H]-inositol phosphate fraction recovered by ion exchange chromatography on Dowex-1 (formate-form columns) and counted. Concentration-response curves for 3 independent experiments performed in duplicate for each mutant are shown in Figure S5. The data show that quisqualate stimulates a concentration-dependent 2-3 fold increase in [^3^H]-inositol phosphate accumulation in HEK293 cells transfected with wild-type human mGluR1. However, none of the mGluR1 mutants showed any significant differences to wild-type with respect to this phospholipase C activity assay, either with respect to the magnitude of the agonist-stimulated increase (E_max_), or the EC_50_ value for quisqualate stimulation.

**
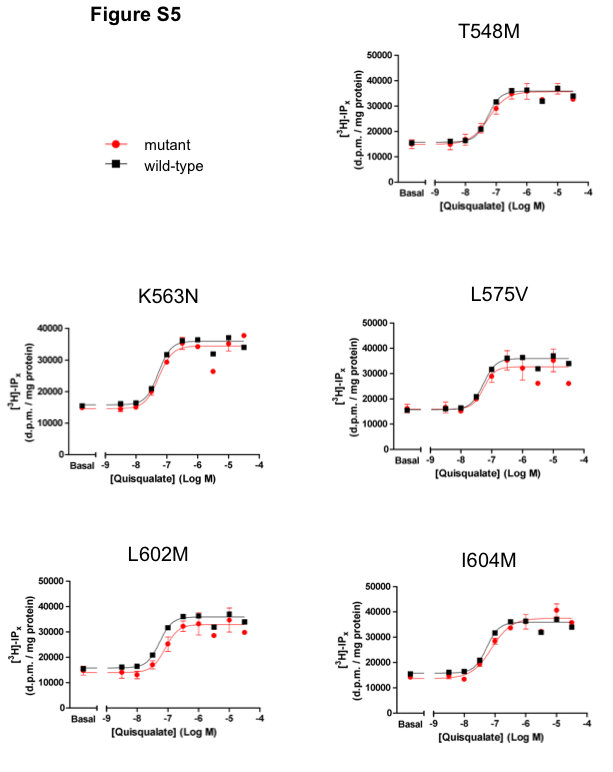
**

**4. TAG-SNP STUDY**

**4.1 Tag-SNP gene prioritization**

The genes used for the tag-SNP study were selected based on proteomic studies of NMDA receptor complexes (NRC) and MAGUK Associated Signaling complexes (MASC)^4,6^. The tag-SNP list included all NRC/MASC genes and additional PSD proteins (see datasheet S6). We included a set of genes that had previously been associated with schizophrenia and other diseases as well as other postsynaptic molecules of potential functional significance in cognitive disorders (datasheet S6).

**4.2 Tag- SNP array design**

To assay the common variation in a gene set for association to disease we selected a set of tagging SNPs (tag-SNPs). tag-SNPs were selected for 265 genes using the Tagger Pair-wise method on HapMap PhaseII CEU data. We aimed to assay 10 kb flanks upstream and downstream of each gene and used the parameters minimum R^2^ of 0.8 and minimum minor allele frequency of 0.05. SNPs were submitted to Illumina for GoldenGate assay design and those judged to be viable assay targets (design score >=0.4) were ordered as 3 Oligo Pool Assays. 12 assays were repeated in all 3 pools as a quality control measure.

**4.3** **Tag-SNP array methods, processing and quality control**

Data processing and quality control was performed as described elsewhere^24^. Briefly, samples were genotyped in 96 well plates; for quality control purposes each plate contained two duplicate samples and one negative (no DNA) control. Illumina software was used to manually inspect the intensity clusters for all SNP assays. Genotypes were assessed using an automated quality control pipeline which identifies poor data; assays with a median GenCall (genotype) score of less than 0.3, greater than 20% missing data or more than one duplicate error per plate were excluded; genotypes with individual GenCall scores less than 0.25 were excluded. Assays not conforming to Hardy-Weinberg were also flagged. A summary of quality control is shown in table S1.

Table S1. Tag-SNP quality control

|  | **Sample quality control** | | | **Assay quality control** | | | **Genotypes** |
| --- | --- | --- | --- | --- | --- | --- | --- |
| OPA | Input samples | Passed  samples | Good genotypes | Input assays | Passed SNPs | Failed SNPs | Genotype released |
| GS0007129 | 1054 | 1054 | 2623389 | 1414 | 1371 | 168 | 2958336 |
| GS0007131 | 1053 | 1052 | 2748463 | 1446 | 1396 | 142 | 3041280 |
| GS0007132 | 1055 | 1053 | 2722765 | 1420 | 1382 | 159 | 3047424 |

Figure S6 shows a Q-Q plot for allelic test of association for tagging SNP dataset. There is no significant deviation from the expected test statistic distribution (dotted line). The inflation factor (lambda=0.98) indicates a lack of population stratification between the samples.


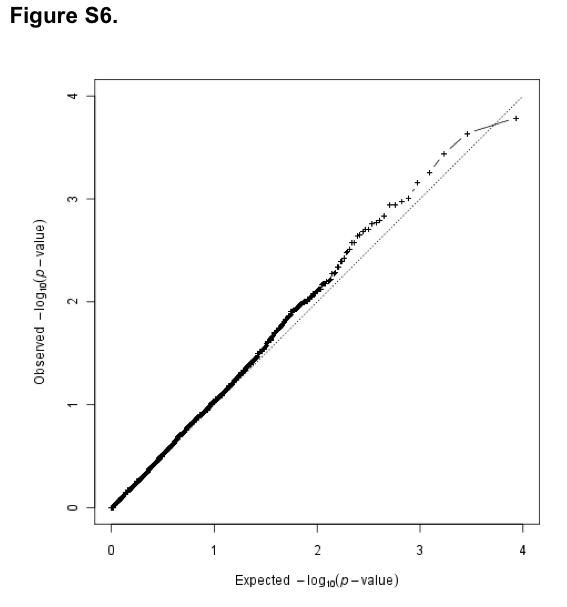


**4.4 Tag-SNP association analysis**

Case-control analyses were performed using a Fisher’s Exact test under both additive and genotypic models. No SNPs remained significant after a Bonferroni correction for the number of SNPs tested. The most significant SNP, rs3105692 in GRIN2A, had a nominal p-value of 0.0002, an order of magnitude greater than the experiment-wide 5% significance cut-off. Twenty-eight SNPs had nominal p-values < 0.005, including multiple SNPs in PRKCE (5), DLG2 (2), GRIN2B (2) and DLGAP1 (2). Twenty-two of the nominally significant SNPs were genotyped in a second independent samples and provided no significant evidence for association,

**4.5 Association by gene**

A whole gene association test statistic was constructed by summing the –log_10_ p-values for all the SNPs within the gene. The significance of this statistic was evaluated using 10,000 permutations, in which case and control labels were randomly shuffled and SNP associations re-calculated (Fisher Exact test under an additive model). Four genes had nominal p-values < 0.01 (PRDX2, GRIN1, RAC1 and CTNNB1). None remained significant after Bonferroni correction for the number of genes tested.

**4.6 Gene rank test**

Genes were ranked by their permutation test p-values (the gene with smallest p-value, PRDX2, having rank = 1). To test whether the 10 hub genes displayed a significant bias towards low ranking, their ranks were summed to provide a test statistic (rank sum = 701). The probability of a random set of 10 genes having a rank sum less than or equal to this value was assessed by generating 100,000 samples of 10 genes.

**4.7 Repeated random sub-sampling validation of genetic load effect**

To test whether total genetic load of variants in the 10 hub genes contributed to susceptibility we employed the following cross-validation procedure:

i) Randomly divide data into discovery (60% individuals) and validation (40%) sets, the ratio of cases to controls being the same in each.

ii) Calculate single SNP association p-values (Fisher Exact test under additive model) in discovery set for all SNPs of the 10 hub genes.

iii) Identify susceptibility alleles (SNPs with p < 0.05).

iv) Calculate total genetic load of susceptibility alleles for each individual in validation set.

v) Perform logistic regression using the model:

Disease status ~ Genotyping rate + Genetic load

where the genotyping rate for each individual is the number of susceptibility alleles successfully genotyped.

vi) Compare to null model (in which only genotyping rate appears), calculating reduction in deviance and (Nagelkerke) pseudo R^2^ associated with full model. The difference in mean genetic load (disease versus control) was also calculated.

Reduction in deviance, R^2^ and difference in mean load were sampled 1000 times using the above procedure. Under the null hypothesis, the reduction in deviance would be expected to follow a chi-square distribution with one degree of freedom. The reduction in deviance from the 1000 cross-validation replications had a mean of 3.27 (P = 0.07). Similarly, the mean difference in genetic load would be expected to follow a normal distribution with mean = 0 and standard deviation = 1. The mean of the sample distribution (mean = 1.23, standard deviation = 0.67) was not significantly different from that expected under the null (χ^2^ = 3.4, P = 0.07). The mean regression coefficient for genetic load from these cross-validation analyses was 0.021, corresponding to a case-control odds-ratio of 1.027 (an increase and effect size of ~3%).

**4.8 Network analysis**

Protein interactions were manually curated from existing datasets^25^ and the UniHi database^26^. We first identified proteins represented in the tag-SNP dataset that directly interact with the 10 glutamatergic ‘hub’ proteins (primary interactors). We then curated all other interactions between tag-SNP proteins and these primary interactors. In total, this identified 267 interactions between 89 proteins (including the 10 hubs) (see datasheet S8). For each protein (excluding the 10 hubs), we then counted the number of hub genes lying with 2 interactions distance (see datasheet S8).

In addition to the analysis reported in the main text, we also asked whether it was possible to identify functional sub-components within glutamate pathways that contribute significantly towards susceptibility. MAGUK-associated signalling complexes (MASCs) are major elements of the glutamatergic ‘machinery’. A study of the MASC interaction network found that when proteins were clustered according to their pattern of connectivity, the clusters possessed identifiable functional roles^25^. Mapping clusters with >10 proteins onto the tag-SNP dataset, the ‘ionotropic input’ cluster containing receptors, scaffolders and closely associated signalling molecules was found to be enriched with low ranking genes (gene rank test P = 0.0098, Figure S7-8). This remained significant after correcting for the number of clusters tested (Bonferroni Step-down P = 0.029). This cluster contained 22 proteins, including 7 of the 10 glutamatergic ‘hub’ genes.

**
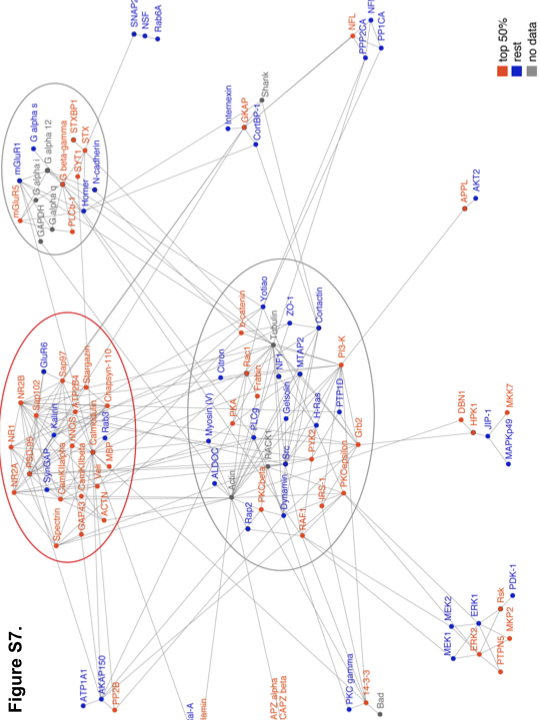
**

**4.9 Results of tag-SNP study**

We performed a parallel genotyping study of 474 schizophrenia cases and 532 controls, examining the disease-association of common alleles from a set of 265 interacting synaptic genes, including the hub genes. Consistent with other genome-wide tag-SNP arrays of large schizophrenic and bipolar cohorts, no significant association for any one allele or gene emerged after correction for multiple testing (see supporting information 3.4-5)^27-29^. However, when genes were ranked by their (permutation test) p-value it was apparent that the 10 glutamatergic hub genes possessed a significant bias towards high rank (rank sum P = 0.0034, see supporting information 3.6) and were thus more associated with disease (Figure S8A). This suggested that multiple common alleles within these genes contributed to an increased genetic load associated with disease. Genetic load was tested by repeated sub-sampling and cross-validation (supporting information 3.7). Susceptibility alleles (SNPs with association p-value < 0.05) were identified in a random 60% of disease and control individuals, tested in the remaining 40%, and was evaluated by logistic regression. From 1000 samples the mean reduction in deviance was 3.27 (mean pseudo R^2^ = 0.01; χ^2^ p-value of 0.07) and the effect size was 3% (see supporting information 3.7). This modest effect provides further evidence that multiple common alleles in these hub genes perhaps contribute to the increased genetic load in disease and is consistent with that found for concurrent nsSNPs of the glutamatergic hub genes.

Next, the possibility was considered that the risk of schizophrenia conferred by common alleles extended beyond the hubs genes to those encoding other components of glutamatergic signalling complexes. Protein-protein interaction data were first curated to identify tag-SNPs within genes that interact with one or more of the 10 hub genes, either directly or via a single intermediary (supporting information 3.8). These were then grouped into successively smaller sets, consisting of genes linked (directly or by a single intermediary) to N or more hub genes (N = 1, 2…8). As N increased, the average ranking of the group was found to decrease (linear regression, P = 0.019, Figure S8B and supporting information 3.8), indicating that proteins more connected to the hubs contributed a higher genetic susceptibility towards disease.


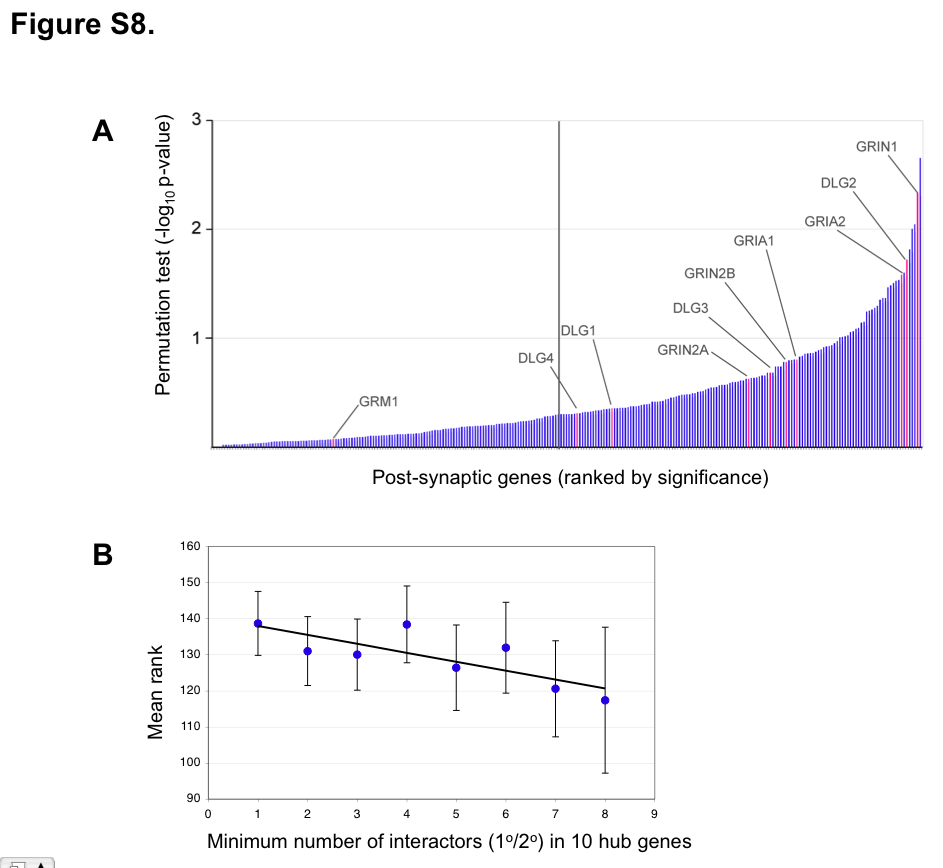


**6.0. LBC COGNITIVE PHENOTYPES**

Association analysis was performed on a range of cognitive ability phenotypes in the Lothian Birth Cohort 1921 (control) samples for both EX and TS studies. No strongly significant associations were observed for any of the traits examined in the EX study. rs7301328 (GRIN2B) was nominally associated with IQ levels at both 11 and 80 years of age (p=0.010 & 0.037 respectively). Nominal associations in GRM1 were observed for the Ravens and logical memory tests at SNPs rs6923492 (p=0.014) and rs2942 (p=0.008) repectively.

In the TS study, once again no strong associations were observed after accounting for the number of SNP-traits combinations tested. SNPs with p-values in the range 0.0001 to 0.005 were observed in all traits examined. Multiple SNPs in GRIN2A and DLGAP1 showed nominally significant results for the IQ measures (IQ at ages 11 and 80, average IQ at these ages and change in IQ between these ages), with the SNPs implicated overlapping only partially between traits. Other genes with multiple nominally significant SNPs for a given trait were DLG2 for IQ at age 80, TTLL3 for logical memory and DISC1 for the Raven’s tests.

As in the schizophrenia analysis of tag-SNPs (see section 3.5 above), a gene base association test combined with permutation (i.e. permuting cognitive phenotype scores between individuals) was used to rank genes in each of the four phenotypes: Moray House Test IQ at11, Moray House Test IQ change between the ages of 11 and 79, RAVENS Standard Progressive Matrices and Wechlser Memory Scale-Revised Logical Memory tests. Very few genes had uncorrected p-values < 0.05, and none remained significant after Bonferroni Step-down correction for multiple testing. No bias in ranking was found for the 10 hub genes. Finally, we tested if any genes had consistently low rankings across all four phenotypes. The rank sum for each gene was calculated, and the probability of obtaining this rank sum by chance was estimated using random sampling (with replacement, since a gene may have the same rank for multiple phenotypes). The degree of correlation between rankings was generally quite low. The top two genes were PRDX2 (mean rank 16.75, P = 0.0002) and DISC2 (mean rank 20, P = 0.0004), although they did not remain significant after correction for multiple testing. Overall, these analyses suggest a much larger cohort may be required to find genetic association between these synaptic gene loci and cognitive performance.

**References**

1. Croning, M.D., Marshall, M.C., McLaren, P., Armstrong, J.D. & Grant, S.G. G2Cdb: the Genes to Cognition database. *Nucleic Acids Res* **37**, D846-51 (2009).

2. Sherry, S.T., Ward, M. & Sirotkin, K. dbSNP-database for single nucleotide polymorphisms and other classes of minor genetic variation. *Genome Res* **9**, 677-9 (1999).

3. Deary, I.J., Whiteman, M.C., Starr, J.M., Whalley, L.J. & Fox, H.C. The impact of childhood intelligence on later life: following up the Scottish mental surveys of 1932 and 1947. *J Pers Soc Psychol* **86**, 130-47 (2004).

4. Husi, H., Ward, M.A., Choudhary, J.S., Blackstock, W.P. & Grant, S.G. Proteomic analysis of NMDA receptor-adhesion protein signaling complexes. *Nature Neuroscience* **3**, 661-9 (2000).

5. Newpher, T. & Ehlers, M. Glutamate Receptor Dynamics in Dendritic Microdomains. *Neuron* **58**, 472-497 (2008).

6. Collins, M. et al. Molecular characterization and comparison of the components and multiprotein complexes in the postsynaptic proteome. *Journal of Neurochemistry* **97**, 16-23 (2006).

7. Ashurst, J.L. et al. The Vertebrate Genome Annotation (Vega) database. *Nucleic Acids Res* **33**, D459-65 (2005).

8. Hubbard, T. et al. The Ensembl genome database project. *Nucleic Acids Res* **30**, 38-41 (2002).

9. Apweiler, R. et al. UniProt: the Universal Protein knowledgebase. *Nucleic Acids Res* **32**, D115-9 (2004).

10. Shen, M., Davis, F. & Sali, A. The optimal size of a globular protein domain: A simple sphere-packing model. *Chemical Physics Letters* **405**, 224-228 (2005).

11. Ng, P.C. & Henikoff, S. Predicting the effects of amino acid substitutions on protein function. *Annual review of genomics and human genetics* **7**, 61-80 (2006).

12. Ramensky, V., Bork, P. & Sunyaev, S. Human non-synonymous SNPs: server and survey. *Nucleic Acids Research* **30**, 3894-900 (2002).

13. Ng, P. SIFT: predicting amino acid changes that affect protein function. *Nucleic Acids Research* **31**, 3812-3814 (2003).

14. Shi, J., Blundell, T.L. & Mizuguchi, K. FUGUE: sequence-structure homology recognition using environment-specific substitution tables and structure-dependent gap penalties. *Journal of Molecular Biology* **310**, 243-57 (2001).

15. Dunbrack, R.L., Jr. & Cohen, F.E. Bayesian statistical analysis of protein side-chain rotamer preferences. *Protein Sci* **6**, 1661-81 (1997).

16. Kleywegt, G.J. Recognition of spatial motifs in protein structures. *J Mol Biol* **285**, 1887-97 (1999).

17. Smith, R.E., Lovell, S.C., Burke, D.F., Montalvao, R.W. & Blundell, T.L. Andante: reducing side-chain rotamer search space during comparative modeling using environment-specific substitution probabilities. *Bioinformatics* **23**, 1099-105 (2007).

18. Worth, C.L. et al. A structural bioinformatics approach to the analysis of nonsynonymous single nucleotide polymorphisms (nsSNPs) and their relation to disease. *Journal of bioinformatics and computational biology* **5**, 1297-318 (2007).

19. Obenauer, J.C., Cantley, L.C. & Yaffe, M.B. Scansite 2.0: Proteome-wide prediction of cell signaling interactions using short sequence motifs. *Nucleic Acids Res* **31**, 3635-41 (2003).

20. Blom, N., Gammeltoft, S. & Brunak, S. Sequence and structure-based prediction of eukaryotic protein phosphorylation sites. *J Mol Biol* **294**, 1351-62 (1999).

21. Cartegni, L., Chew, S.L. & Krainer, A.R. Listening to silence and understanding nonsense: exonic mutations that affect splicing. *Nature Reviews Genetics* **3**, 285-98 (2002).

22. Woolfe, A., Mullikin, J.C. & Elnitski, L. Genomic features defining exonic variants that modulate splicing. 1-23.

23. Aricescu, A., Lu, W. & Jones, E. A time- and cost-efficient system for high-level protein production in mammalian cells. *Acta Crystallographica Section D Biological Crystallography* **62**, 1243-1250 (2006).

24. Brown, W.M. et al. Overview of the MHC fine mapping data. *Diabetes Obes Metab* **11 Suppl 1**, 2-7 (2009).

25. Pocklington, A., Cumiskey, M., Armstrong, J. & Grant, S. The proteomes of neurotransmitter receptor complexes form modular networks with distributed functionality underlying plasticity and behaviour. *Molecular Systems Biology* **2**, 14 (2006).

26. Chaurasia, G. et al. UniHI: an entry gate to the human protein interactome. *Nucleic Acids Res* **35**, D590-4 (2007).

27. Need, A. et al. A Genome-Wide Investigation of SNPs and CNVs in Schizophrenia. *PLoS genetics* **5**, e1000373 (2009).

28. Sklar, P. et al. Whole-genome association study of bipolar disorder. *Molecular psychiatry* **13**, 558-569 (2008).

29. Sullivan, P.F. et al. Genomewide association for schizophrenia in the CATIE study: results of stage 1. *Molecular psychiatry* **13**, 570-84 (2008).
